# Screening Performance for Insufficient Vegetable Intake in Young Adult Japanese Women: Spot Urinary Potassium Excretion as a Simple, Non-Invasive Marker

**DOI:** 10.3390/nu18050735

**Published:** 2026-02-25

**Authors:** Asaki Mizutani, Marina Yamagishi, Mami Sakuda, Ayuka Mita, Emi Morita, Naoko Suga, Atsuko Kitano, Rika Ohara, Akira Takamata, Junko Ishihara, Ribeka Takachi

**Affiliations:** 1 Department of Food Science and Nutrition, Nara Women’s University Graduate School of Humanities and Sciences, Kitauoya-Higashimachi, Nara-shi, Nara 630-8506, Japan; 2 Graduate School of Public Health, International University of Health and Welfare, 4-1-26 Akasaka, Minato-ku, Tokyo 107-8402, Japan; 3 Graduate School of Environmental Health, Azabu University, 1-17-71 Fuchinobe, Chuo-ku, Sagamihara-shi, Kanagawa 252-5201, Japan

**Keywords:** vegetables, urinary potassium, spot urine, mass screening

## Abstract

**Background/Objectives:** Existing methods for assessing the intake of vegetables and fruits have limitations. This study aims to investigate the number of measurements required to screen individuals with insufficient usual vegetable intake or total fruit and vegetable intake using urinary potassium excretion estimated from spot urine samples, a non-invasive and simple biomarker. **Methods:** A total of 97 women aged 18–24 years provided a 12-day dietary survey, four 24 h urine collections, and three timed spot urine samples per day, collected in a duration of 4 days. Using the dietary surveys as a reference, we evaluated the number of spot urine samples required and their screening performance for identifying individuals whose vegetable intake or total fruit and vegetable intake was below the study population mean. Screening performance was assessed by receiver operating characteristic analysis and the area under the curve (AUC). **Results:** For vegetable intake, the AUCs were >0.70 when either a single spot urine sample was collected at the second urine void after waking or ≥2 spot urine samples were collected at arbitrary time points. For total fruit and vegetable intake, AUCs were >0.70 when using a single spot urine sample collected at the second void after waking up. **Conclusions:** These findings suggest that a time-specific single spot urine sample (i.e., the second urine void after waking) or ≥2 spot urine samples collected at arbitrary time points may help in screening individuals with habitually insufficient vegetable intake.

## 1. Introduction

Vegetable and fruit consumption is associated with various health outcomes, including all-cause mortality, cancer and cardiovascular disease [1,2,3]. To promote sufficient consumption, many countries have established certain guidelines and dietary goals [4,5]. In Japan, for instance, the Basic Direction for Comprehensive Implementation of National Health Promotion (Health Japan 21, the Third Term) recommends an intake of 350 g of vegetables and 200 g of fruit per day [6]; however, the most recent national survey (2023) reported mean intakes of 256 g for vegetables and 93 g for fruit (212 g and 48 g, respectively, among women in their 20s) [7]. It is, therefore, important to assess whether individuals meet target intake levels. When the overall intake of a population falls below national recommendations, a distribution-based approach may be more practical. This approach helps identify priority groups with relatively low vegetable and/or fruit intake.

Various methods have been used to assess vegetable and fruit intake. However, each has limitations. Dietary assessments based on self-reporting are subject to measurement errors owing to memory bias and recording burden [8,9,10]. Blood carotenoids and vitamin C are invasive biomarkers. Conversely, skin carotenoid measurements can be measured in a non-invasive manner using spectrophotometry techniques such as resonance Raman spectroscopy, reflection spectroscopy, and spectrophotometry [11,12]. However, previous studies examining the criterion-related validity of these methods have been limited to measures of association, such as correlation coefficients (CCs) and linear regression analysis [13]. Furthermore, urinary flavonoids when measured as a single phenolic compound cannot adequately reflect total fruit and vegetable intake [8].

Vegetables and fruits are the major sources of potassium intake among multiple sources such as potatoes, grains, milk and dairy products, and meat [7,14,15]. Some studies have explored the use of urinary potassium as a potential biomarker of vegetable and fruit consumption [16,17,18,19]. These studies have relied on 24 h urine collections (24 h UCs). A previous study investigated whether potassium excretion measured in a single 24 h UC could identify individuals with insufficient usual vegetable intake and found that it achieved acceptable screening performance [16]. However, the 24 h UC is cumbersome and burdensome for participants, potentially resulting in incomplete collection [8], thus having practical limitations. Accordingly, measuring potassium in spot urine samples may be a convenient method for assessing vegetable and fruit intake. This study aimed to investigate the number of measurements required and the screening performance of urinary potassium excretion estimated from spot urine samples for identifying individuals with insufficient usual vegetable intake or total fruit and vegetable intake (below target levels and mean levels).

## 2. Materials and Methods

### 2.1. Study Setting and Participants

This study was conducted between July 2024 and May 2025 among female university students aged 18–24 years enrolled at Nara Women’s University, Japan. Individuals who regularly took medication for any disease were excluded from recruitment. Participants were recruited using promotional materials such as flyers. Following a detailed explanation of the study during lunchtime, written informed consent was obtained from 120 individuals. At the end of the study, participants received a monetary compensation and obtained a summary of their 12-day dietary survey, as well as their urine and blood measurement results and nutrition-related messages. This study was conducted in accordance with the Declaration of Helsinki and approved by the Institutional Review Boards of the Ethics Review Committee of Nara Women’s University, Nara, Japan (No. 25–43), and of Azabu University, Kanagawa, Japan (No. 160).

### 2.2. Data Collection

**Figure 1:**
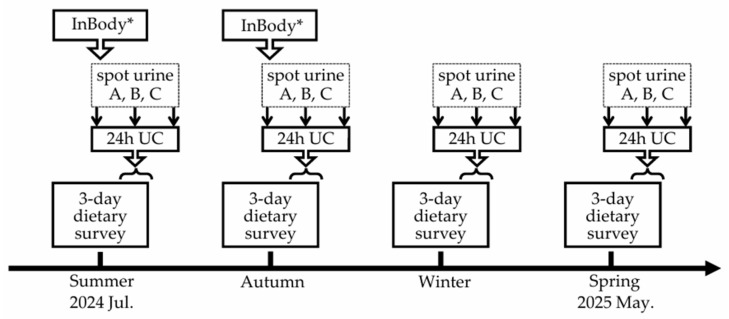
Study schedule. Abbreviations: InBody, assessment of body weight using a body composition analyzer (InBody 380 N, InBody Co., Ltd., Seoul, Republic of Korea), and body height using a stadiometer; spot urine A, B, C, spot urine samples collected at three time points: (A) the second urine void after waking; (B) afternoon sample; (C) first urine void on the following morning; 24 h UC, 24 h urine collection; 3-day dietary survey, 3-day dietary survey using a mobile-based photographic dietary record system. * Conducted in July for 60 participants [61.9%] and in October for 37 participants [38.1%].

The study schedule is shown in Figure 1. Between July 2024 and May 2025, participants were asked to complete a 12-day dietary survey, four seasonal 24 h UCs, and spot urine collections during the same day (three samples: the second urine void after waking, one at an arbitrary time point in the afternoon, and the first urine void on the following morning). For the 12-day dietary survey, participants recorded their meals by using a mobile-based photographic dietary record system over a duration of 3 days per season (approximately once every 3 months), regardless of whether the days were consecutive. The 24 h UC and the corresponding three spot urine samples were self-administered on the last day of each 3-day dietary survey period (from the second urine void on the last survey day to the first urine void on the following morning). Body weight was measured using a body composition analyzer (InBody 380 N, InBody Co., Ltd., Seoul, Republic of Korea), and body height was measured using a stadiometer in July (*n* = 60; 61.9%) or October (*n* = 37; 38.1%) of 2024. Age information was collected via self-report at the time of first entry in the dietary record system. Of the 120 participants who provided written informed consent, 23 (19.2%) withdrew before or during the study period; the remaining 97 participants completed the survey.

### 2.3. 12-Day Dietary Survey

For the 12-day dietary survey, participants recorded their meals using the mobile-based photographic dietary record system. This system’s user interface was designed to allow participants to self-administer their dietary records. Following on-screen instructions, participants recorded their meals by uploading photographs of each dish taken with a pre-provided reference scale and selecting the corresponding dish names and portion sizes from the system’s recipe database [20,21,22]. This system serves as the successor to the PC version, allowing real-time recording of dish photographs. Participants were asked to complete these records either during the meal or on the same day as the meal. That is, although they were permitted to register everything later in bulk, they had to take photographs of their dish every time they ate or drank. Dietary records were corrected for consistency with photographs by trained investigators and, if necessary, reviewed remotely via online chat on the weekday following recording. Subsequently, investigators edited the built-in recipe data, including individual ingredients and weights. Nutritional values were calculated using the weight of each item and the Standard Tables of Food Composition in Japan 2020 (FCT) [23].

### 2.4. Urine Collection

For the 24 h UC, participants used a portable urine measurement device (Urine Mate P, Sumitomo Bakelite Co., Ltd., Tokyo, Japan), which collects 1/50 of the volume from each void. At designated time points, spot urine samples were collected from the remaining urine. Samples were stored in a cool, dark location after collection and placed in a deep freezer (−80 °C) on the day urine collection was completed. Urinary potassium concentration (mEq/L) was measured using the ion-selective electrode method, and urinary creatinine concentration (mg/dL) was measured using an enzymatic method at Kotobiken Medical Laboratories Inc. (Tokyo, Japan). In cases where a single collection error occurred (e.g., a missed void or accidental spillage), the total urinary volume was corrected by substituting the mean volume calculated from successfully obtained urine collections. If ≥2 errors occurred, the respective 24 h UC was excluded from the analysis. Most participants completed all four 24 h UCs with fewer than two errors: 95% completed four collections, and 5% completed three. For the second spot urine sample, 78%, 19%, 1%, and 2% completed four, three, two, and one collection, respectively. For the afternoon spot urine sample, 93%, 4%, and 3% completed four, three, and two collections, respectively. For the first spot urine sample on the following morning, 93%, 6%, and 1% of participants completed four, three, and two collections, respectively. In cases where measurements were not available for all intended seasons or samples, mean values were calculated based on available data. Urinary potassium excretion (mg/day) was calculated directly from 24 h UCs and estimated from spot urine samples using the Kawasaki or Tanaka equations [24,25], as shown below. In both equations, predicted 24 h urinary creatinine excretion was calculated based on age, sex, height, and body weight, according to the original methods [25,26]. Considering that approximately 77% of potassium intake is excreted in urine [8,27], it was estimated by dividing urinary potassium excretion by 0.77.

$$\begin{array}{c} Potassium\;excretion\;from\;24-h\;UCs\;(mg/day):\\ \;\;\;\;\;\;\;\;\;collected\;urinary\;volume\;\left(mL\right)\times 50/1000\times potassium\;(mEq/L)\times 39;\\ Potassium\;excretion\;using\;the\;Kawasaki\;equation\;(mg/day):\\ \;\;\;\;\;\;\;\;\;\;\;\;\;\;\;\;\;\;7.2\times {\left(potassium\;(mEq/L)\;/\;creatinine\;(mg/dL)/10\times predicted\;creatinine\;(mg/day)\right)}^{0.5}\times 39,\\ predicted\;creatinine:\\ \;\;\;\;\;\;-4.72\times Age+8.58\times Weight\;(kg)+5.09\times Height\;(cm)-74.95;\\ Potassium\;excretion\;using\;the\;Tanaka\;equation\;(mg/day):\\ \;\;\;\;\;\;\;\;\;\;\;\;\;\;\;\;7.59\times {\left(potassium\;(mEq/L)\;/\;creatinine\;(mg/dL)/10\times predicted\;creatinine\;(mg/day)\right)}^{0.431}\times 39,\\ predicted\;creatinine:\\ -2.04\times Age+14.89\times Weight\;(kg)+16.14\times Height\;(cm)-2244.45. \end{array}$$

### 2.5. Statistical Analysis

Analyses were conducted only on the 97 participants who completed the study. Pearson’s CCs were calculated to assess the associations between usual intakes (potassium, vegetables, or total fruit and vegetables) and urinary potassium excretion obtained from 24 h UCs or spot urine samples using either the Kawasaki or the Tanaka equations, after performing log transformation of all variables. For 24 h UC measurements, annual means were determined by averaging the measurements across all four seasons or randomly selected one to three seasons; these values were used for comparison with spot urine-based estimates. For estimates based on the Kawasaki equation, designed for use with the second urine void after waking, a single spot urine sample per season was used, and annual means were determined by averaging the estimates across all four seasons or randomly selected one to three seasons. The Tanaka equation, applicable to spot urine samples collected at arbitrary time points, was applied to up to three spot urine samples per season. For this equation, annual means were determined by averaging values based on three or randomly selected one to two spot urine samples per season and then averaging these values across all four seasons or randomly selected one to three seasons. In addition, the Tanaka equation was also applied to a second urine void after waking to determine whether differences in screening performance were attributable to the choice of equation rather than to differences in urine sampling. These analytic patterns were applied consistently across all CCs and receiver operating characteristic (ROC) analyses.

ROC analysis was then performed to evaluate the screening performance of urinary potassium excretion estimated from spot urine samples collected across four seasons. The analysis examined the ability of these estimates to identify individuals with insufficient usual vegetable intake or total fruit and vegetable intake; the areas under the curve (AUCs) and their 95% confidence intervals (CIs) were calculated. The mean intake estimated from the 12-day dietary survey over 1 year was used as the reference standard. Individuals were classified into sufficient/insufficient intake groups for vegetable intake or total fruit and vegetable intake according to predefined intake criteria (≥150 to ≥350 g/day in 50 g/day increments). Sensitivity was defined as the proportion of participants whose urinary potassium excretion levels were below the corresponding threshold among those whose vegetable intake or total fruit and vegetable intake was classified as insufficient. Specificity was defined as the proportion of participants whose urinary potassium excretion levels were above the corresponding threshold among those whose vegetable intake or total fruit and vegetable intake was classified as sufficient. The optimal cutoff values of urinary potassium excretion were determined by calculating sensitivity and specificity across a range of possible thresholds obtained for drawing the ROC curve. The value that maximized specificity among those with a sensitivity >0.80 was selected. This approach aligns with prior recommendations for screening test design, which emphasize maintaining sufficiently high sensitivity to reduce false-negative results [28]. Screening performance was considered acceptable for AUC > 0.70 and a lower bound of 95% CI > 0.50 [16,29].

For primary analysis, we examined the number of spot urine samples required per participant to achieve acceptable screening performance by evaluating the screening performance of averaged urinary potassium excretion values. These values were estimated from randomly selected subsets of seasonal spot urine samples. In this analysis, considering the intake distribution in the target age group, approximate mean values derived from the 12-day dietary survey (≥150 g/day for vegetables and ≥200 g/day for total fruit and vegetables) were used as intake criteria. Since the results remained mostly unchanged irrespective of including or excluding participants with incomplete urine collections, the results for all participants included are presented. All analyses were performed using SAS version 9.4 (SAS Institute Inc., Cary, NC, USA).

## 3. Results

Table 1 shows participants’ characteristics. The mean energy intake was 1440 kcal. The mean intake values for potassium, total fruit and vegetables, vegetables, and fruit were 1661 mg, 206 g, 155 g, and 51 g/day, respectively, based on the 12-day dietary survey. The mean urinary potassium excretion measured from four 24 h UCs was 1338 mg; considering that ~77% of the intake is excreted [8,27], the estimated potassium intake was 1737 mg/day.

Table 2 shows Pearson’s CCs between the usual intake of potassium, vegetables, or total fruit and vegetables, and urinary potassium excretion from 24 h UCs or spot urine samples. The CCs between potassium intake based on the 12-day dietary survey and potassium excretion based on four 24 h UCs were 0.53. Based on estimates from spot urine samples collected across 4 days, the CCs were found to range moderately from 0.40 to 0.55 for potassium intake, 0.37 to 0.43 for vegetable intake, and 0.39 to 0.46 for total fruit and vegetable intake. The CCs, based on 4-day collection, compared with urinary potassium excretion measured from 24 h UCs were 0.63 for the Kawasaki equation, 0.75 for the Tanaka equation using three spot urine samples, 0.69 using two samples, and 0.62 using one sample.

Acceptable screening performance was achieved for vegetable intake and total fruit and vegetable intake using urinary potassium excretion estimated from spot urine samples averaged across 4 days when intake criteria were set near the study population mean (i.e., ≥150 g/day for vegetables and ≥200 g/day for total fruit and vegetables), with AUCs > 0.70 and the lower limit of 95% CIs > 0.50 (Table 3). This screening performance was consistent irrespective of the number of samples per day or collection timing. The corresponding optimal cutoff values of urinary potassium excretion for vegetable intake were 1820 mg/day, as determined using the Kawasaki equation, and 1374 mg/day using the Tanaka equation, based on three spot urine samples. Corresponding values for total fruit and vegetable intake were 1851 and 1383 mg/day, respectively. At higher intake criteria (≥300 g/day and ≥350 g/day, Table S1), almost all participants were classified below the thresholds, resulting in a marked imbalance between the two groups. In this context, based on the Tanaka equation estimates, the AUCs > 0.70 were observed for vegetable intake. For fruit intake, the screening performance of urinary potassium excretion, measured from four 24 h UCs, was below the acceptable level (AUC = 0.58) at an intake criterion of ≥50 g/day, which was about the study population mean intake.

Table 4 shows the number of urine collection days required and the corresponding screening performance of urinary potassium excretion, obtained from 24 h UCs or spot urine samples, for identifying individuals with insufficient usual vegetable intake (≥150 g/day) or total fruit and vegetable intake (≥200 g/day). For vegetable intake, an acceptable screening performance (AUC > 0.70) was achieved using the Kawasaki equation based on a single second urine after waking and the Tanaka equation based on ≥2 spot urine samples collected at arbitrary time points within a single day. The single 24 h UC slightly fell below the acceptable level (AUC = 0.69). When the Tanaka equation was applied using only one spot urine sample per day collected at an arbitrary time point, AUC values fell slightly below the acceptable level when only 1- or 2-day collections were obtained (AUC = 0.67 and 0.68, respectively) despite the lower limits of the 95% CIs exceeding 0.50. For total fruit and vegetable intake, acceptable screening performance was achieved using either the Kawasaki equation based on a single second urine sample or the Tanaka equation based on either 2-day collection of three spot urine samples per day or 3-day collections of two spot urine samples per day. In contrast, the AUC value fell below the acceptable level even for 4-day collections of one spot urine sample per day collected at an arbitrary time point. When the Tanaka equation was applied to a single second urine after waking, acceptable screening performance was observed with fewer collections than when samples were collected at arbitrary time points; specifically, acceptable AUC values were achieved with 1-day collection for both vegetable intake and total fruit and vegetable intake.

**Figure 2:**
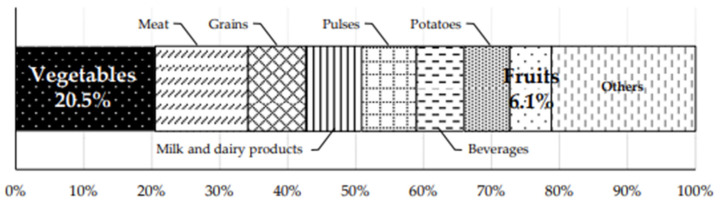
Relative contributions of food groups to potassium intake based on a 12-day dietary survey using a mobile-based photographic dietary record system.

Regarding the potassium sources from the food groups shown in Figure 2, vegetables accounted for the largest proportion at 20.5%, whereas fruits accounted for 6.1%.

## 4. Discussion

The present results indicate that, for vegetable intake, either a single spot urine sample collected at the second urine void after waking or ≥2 spot urine samples collected at arbitrary time points within a day achieved acceptable performance to screen individuals with usual vegetable intake below the study population mean. For total fruit and vegetable intake, acceptable screening performance was achieved using a single spot urine sample collected at the second void after waking.

In the present study, potassium excretion estimated from 4-day spot urine samples showed correlations of 0.37–0.46 with usual vegetable intake and total fruit and vegetable intake. These values are comparable to those previously reported in studies using blood carotenoids, blood vitamin C, and skin carotenoids as biomarkers of vegetable intake and total fruit and vegetable intake (0.22–0.48) [8,13]. Therefore, although urinary potassium lacks specificity for vegetables, spot urinary potassium may be a useful non-invasive, simple biomarker for assessing vegetable intake and total fruit and vegetable intake. Furthermore, high correlations were observed between urinary potassium excretion measured from four 24 h UCs and the estimated urinary potassium excretion derived from spot urine samples collected across 4 days (r = 0.62–0.75), which further supports the reliability of spot urinary potassium measurements as surrogate marker for vegetable consumption and total fruit and vegetable consumption.

Furthermore, our results suggest that even a single spot urine sample for potassium measurement collected at an appropriate time point can achieve acceptable screening performance. A previous study reported that individuals with insufficient usual vegetable intake could be identified with acceptable screening performance using a single 24 h UC (AUC = 0.77 for men and 0.71 for women) among middle-aged and older adults [16]. In this study, spot urine samples had a similar screening performance to that of 24 h UC, highlighting their potential as a simple alternative for population-level screening. Furthermore, AUCs were comparable across different numbers of urine collection days, with overlapping 95% confidence intervals. Accordingly, the absence of a significant difference across collection frequencies suggests that the screening performance of urinary potassium remains acceptable even with a reduced number of collection days.

Our findings suggest that a higher number of single spot urine samples collected at arbitrary time points may be required to screen usual vegetable intake or total fruit and vegetable intake compared with spot urine samples collected at a specific time point, such as the second urine void after waking. This may be partly explained by the known diurnal variation in urinary potassium excretion [30]. In a previous study, urinary potassium excretion estimated from spot urine samples collected at the second void after waking or in the afternoon was more strongly correlated with urinary potassium excretion measured by 24 h UC [31]. Accordingly, the Kawasaki equation adopts potassium concentrations from the second urine void after waking [24]. Consistently, in our study, spot urine samples collected at the second void after waking performed better than those collected at arbitrary time points.

For total fruit and vegetable intake, a higher number of spot urine samples was required to achieve acceptable screening performance compared with vegetable intake alone. Moreover, for fruit intake alone, acceptable screening performance was not achieved even when four 24 h UCs were used. Therefore, urinary potassium cannot be considered a sufficient screening indicator for fruit intake alone; thus, it should not be overinterpreted as an indicator of fruit consumption. This may be owing to a higher day-to-day variation in the intake of fruits compared to vegetables [16] and/or a relatively low contribution of fruit to overall potassium intake. In this study, vegetables accounted for the largest proportion of total potassium intake among food groups (20.5%), whereas fruits accounted for 6.1%, ranking eighth. These findings are consistent with those reported in the National Health and Nutrition Survey in Japan in 2023 with corresponding values of 21.5% and 7.9%, respectively [7].

This study has several limitations. First, the dietary survey using the dish photographic record system used as the reference differed from commonly used high-precision methods such as the weighed food record (WFR); thus, the estimated intake values may contain measurement errors. The lack of a direct examination of the validity of vegetable and fruit intake measured by this system is a potential limitation, although investigators verified the registered information based on real-time dish photographs. The CC between potassium intake and urinary potassium excretion measured from four 24 h UCs in this study (r = 0.53) was slightly lower than that reported in a previous study among Japanese young women using WFR (r = 0.58) [32]. Therefore, the screening performance of urinary potassium excretion may have been underestimated owing to measurement errors in the dietary survey used as a reference standard. Second, the fact that vegetable intake used as a reference criterion may have been underestimated owing to potential differences in dietary survey methods rather than differences in group characteristics cannot be ruled out. This interpretation is supported by the fact that the mean potassium intake estimated from four 24 h UCs (1737 mg) was comparable to, or at most approximately 5% lower than, those reported in the National Health and Nutrition Survey in Japan in 2023 for women in their 20s (1830 mg) [7], whereas the mean vegetable intake in the present study (155 g) was approximately 27% lower than that observed in the national survey (212 g) [7]. In the present study, vegetables contributed a similar proportion of total potassium intake (20.5%) as reported in the national survey (21.5%) [7]. In addition, the major contributing food groups were consistent between the two datasets. Third, study participants only included young women from a specific region in Japan. Therefore, caution is warranted when generalizing the findings to populations with different characteristics, such as place of residence, age, sex, or overall intake level. Differences in these characteristics may alter the proportion of potassium derived from vegetables and fruits. A higher proportion may improve the screening performance for urinary potassium, whereas a lower proportion may reduce it. Fourth, the study did not fully evaluate the screening performance achieved to try and discern whether individuals met the national recommended intake levels for vegetables and fruits, because the participants were distributed in the low intake range relative to these levels. When applying this screening approach using national targets as intake criteria, diagnostic accuracy may decrease in populations whose intake substantially deviates from these targets.

## 5. Conclusions

This study suggests that, for vegetable intake, either a single spot urine sample collected at the second urine void after waking or ≥2 spot urine samples collected at arbitrary time points within a day achieved acceptable performance to screen individuals with vegetable intake below the mean level. Accordingly, a time-specific single spot urine sample (i.e., the second urine void after waking) or ≥2 spot urine samples collected at arbitrary time points may help in screening individuals with habitually insufficient vegetable intake. Our findings are based on a relatively small and homogeneous study sample; thus, further validation in larger and more diverse populations is needed.

## Figures and Tables

**Table 1:** Characteristics of participants (*n* = 97).

	Mean	SD	Median
Age (years)	19	1	19
Body weight (kg)	51.2	6.6	50.2
Body height (cm)	158.5	5.5	158.2
BMI (kg/m^2^)	20.3	2.0	20.4
12-day Dietary Survey *			
Total energy (kcal)	1440	220	1449
Potassium (mg)	1661	414	1667
Total fruit and vegetables (g)	206	78	196
Vegetables (g)	155	57	152
Fruit (g)	51	39	38
24 h UC			
Urinary potassium excretion (mg) ^†^	1338	433	1305
Adjusted to potassium intake (mg) ^‡^	1737	563	1695
Kawasaki ^§^ Second spot urine after waking (mg)	1786	330	1709
Tanaka^||^ 3 All three spot urine samples (mg)	1351	176	1342
Tanaka^||^ 2 Randomly selected two of three samples (mg)	1344	181	1339
Tanaka^||^ 1 Randomly selected one of three samples (mg)	1335	215	1321
Tanaka^||^ 1 Second spot urine after waking (mg)	1423	248	1378

Abbreviations: SD, standard deviation; BMI, body mass index; 24 h UC, 24 h urine collection. * Intakes estimated from a 12-day dietary survey using a mobile-based photographic dietary record system. ^†^ Measured from four 24 h UCs. ^‡^ Estimated from urinary potassium excretion measured from four 24 h UCs by dividing urinary potassium excretion by 0.77. ^§^ Urinary potassium excretion estimated using the Kawasaki equation. ^||^ Urinary potassium excretion estimated using the Tanaka equation.

**Table 2:** Correlation coefficients between usual intake of potassium, vegetables, or total fruit and vegetables and urinary potassium excretion from 1 to 4 day 24 h UCs or spot urine samples (*n* = 97).

		Usual Intake *		
	Days	Potassium	Vegetables	Total Fruit and Vegetables
24 h UC	4	0.53	0.36	0.37
	3	0.51	0.34	0.35
	2	0.44	0.37	0.36
	1	0.37	0.23	0.28
Kawasaki ^†^	4	0.49	0.39	0.46
Second spot urine after waking	3	0.44	0.32	0.41
	2	0.47	0.31	0.37
	1	0.20	0.27	0.32
Tanaka ^‡^ 3	4	0.55	0.43	0.45
All three spot urine samples	3	0.51	0.37	0.40
	2	0.42	0.38	0.39
	1	0.44	0.35	0.34
Tanaka ^‡^ 2	4	0.51	0.37	0.41
Randomly selected two of three samples	3	0.47	0.37	0.40
	2	0.43	0.33	0.30
	1	0.42	0.30	0.26
Tanaka ^‡^ 1	4	0.40	0.42	0.39
Randomly selected one of three samples	3	0.49	0.31	0.35
	2	0.33	0.29	0.27
	1	0.32	0.25	0.25
Tanaka ^‡^ 1	4	0.47	0.40	0.46
Second spot urine after waking	3	0.45	0.38	0.43
	2	0.37	0.39	0.39
	1	0.38	0.32	0.38

All values were log-transformed before calculation of Pearson’s correlation coefficients. Abbreviations: 24 h UC, 24 h urine collection. * Mean intake estimated from a 12-day dietary survey using a mobile-based photographic dietary record system. ^†^ Urinary potassium excretion estimated using the Kawasaki equation. ^‡^ Urinary potassium excretion estimated using the Tanaka equation.

**Table 3:** AUC (95% CI) of ROC curves for screening individuals with insufficient usual vegetable intake or total fruit and vegetable intake using urinary potassium excretion from 24 h UCs or spot urine samples across 4 days (below target levels and mean levels; *n* = 97).

		Vegetables						Total Fruit and Vegetables						
	Criteria	*n* *	AUC	95% CI	CO ^†^	Se	Spe	Criteria	*n* *	AUC	95% CI	CO ^†^	Se	Spe
24 h UC	≥150 g	47	0.74	0.64–0.84	1423	0.83	0.56	≥200 g	52	0.71	0.60–0.81	1485	0.83	0.49
	≥350 g	96	0.48	0.38–0.58	2983	1.00	0.00	≥350 g	93	0.68	0.41–0.96	1783	0.88	0.50
Kawasaki ^‡^	≥150 g	47	0.75	0.65–0.85	1820	0.81	0.58	≥200 g	52	0.74	0.64–0.84	1851	0.81	0.58
Second spot urine after waking	≥350 g	96	0.59	0.49–0.69	2640	1.00	0.00	≥350 g	93	0.63	0.37–0.90	2180	0.87	0.25
Tanaka ^§^ 3	≥150 g	47	0.78	0.69–0.87	1374	0.83	0.62	≥200 g	52	0.76	0.67–0.86	1383	0.81	0.60
All three spot urine samples	≥350 g	96	0.79	0.71–0.87	1808	1.00	0.00	≥350 g	93	0.66	0.31–1.00	1482	0.81	0.75
Tanaka ^§^ 2	≥150 g	47	0.75	0.65–0.84	1379	0.81	0.54	≥200 g	52	0.72	0.62–0.82	1402	0.83	0.47
Randomly selected two of three samples	≥350 g	96	0.86	0.80–0.93	1536	0.86	1.00	≥350 g	93	0.57	0.20–0.94	1519	0.84	0.50
Tanaka ^§^ 1	≥150 g	47	0.73	0.63–0.83	1397	0.83	0.50	≥200 g	52	0.69	0.58–0.80	1417	0.81	0.42
Randomly selected one of three samples	≥350 g	96	0.94	0.89–0.99	1636	0.94	1.00	≥350 g	93	0.72	0.30–1.00	1596	0.90	0.75
Tanaka ^§^ 1	≥150 g	47	0.76	0.67–0.86	1413	0.83	0.66	≥200 g	52	0.75	0.65–0.85	1486	0.83	0.51
Second spot urine after waking	≥350 g	96	0.73	0.64–0.82	2028	1.00	0.00	≥350 g	93	0.62	0.35–0.90	2028	1.00	0.00

Abbreviations: AUC, area under the curve; CI, confidence interval; ROC, receiver operating characteristic; 24 h UC, 24 h urine collection; CO, cutoff value; Se, sensitivity; Spe, specificity. * Number of participants who deviate from the criterion based on a 12-day dietary survey using a mobile-based photographic dietary record system as the reference. ^†^ Cutoff values determined as thresholds maximizing specificity among those with a sensitivity >0.80. ^‡^ Urinary potassium excretion estimated using the Kawasaki equation. ^§^ Urinary potassium excretion estimated using the Tanaka equation.

**Table 4:** AUC (95% CI) of ROC curves for screening individuals with insufficient usual vegetable intake or total fruit and vegetable intake using urinary potassium excretion from 1 to 4 day 24 h UCs or spot urine samples (*n* = 97).

		Vegetables (Criterion ≥ 150 g, n* = 47)					Total Fruit and Vegetables (Criterion ≥ 200 g, n* = 52)				
	Days	AUC	95% CI	CO^†^	Se	Spe	AUC	95% CI	CO ^†^	Se	Spe
24 h UC	4	0.74	0.64–0.84	1423	0.83	0.56	0.71	0.60–0.81	1485	0.83	0.49
	3	0.71	0.61–0.81	1409	0.83	0.56	0.69	0.58–0.79	1500	0.81	0.47
	2	0.72	0.61–0.82	1374	0.85	0.52	0.71	0.61–0.82	1364	0.81	0.56
	1	0.69	0.58–0.79	1598	0.83	0.32	0.68	0.57–0.79	1671	0.85	0.33
Kawasaki ^‡^	4	0.75	0.65–0.85	1820	0.81	0.58	0.74	0.64–0.84	1851	0.81	0.58
Second spot urine after waking	3	0.73	0.63–0.83	1871	0.83	0.58	0.73	0.63–0.84	1871	0.81	0.60
	2	0.71	0.61–0.81	2025	0.81	0.40	0.71	0.60–0.81	2025	0.83	0.44
	1	0.70	0.59–0.81	1923	0.81	0.46	0.70	0.59–0.81	1923	0.81	0.49
Tanaka ^§^ 3	4	0.78	0.69–0.87	1374	0.83	0.62	0.76	0.67–0.86	1383	0.81	0.60
All three spot urine samples	3	0.73	0.63–0.83	1393	0.81	0.60	0.71	0.61–0.82	1415	0.81	0.47
	2	0.76	0.67–0.86	1376	0.81	0.54	0.71	0.60–0.81	1395	0.81	0.49
	1	0.72	0.62–0.82	1430	0.81	0.50	0.66	0.55–0.77	1487	0.81	0.31
Tanaka ^§^ 2	4	0.75	0.65–0.84	1379	0.81	0.54	0.72	0.62–0.82	1402	0.83	0.47
Randomly selected two of three samples	3	0.73	0.63–0.83	1407	0.81	0.52	0.72	0.62–0.82	1435	0.87	0.47
	2	0.73	0.63–0.83	1460	0.87	0.46	0.68	0.57–0.78	1518	0.81	0.33
	1	0.75	0.65–0.85	1386	0.87	0.52	0.68	0.57–0.79	1468	0.87	0.42
Tanaka ^§^ 1	4	0.73	0.63–0.83	1397	0.83	0.50	0.69	0.58–0.80	1417	0.81	0.42
Randomly selected one of three samples	3	0.73	0.63–0.83	1391	0.81	0.54	0.70	0.60–0.81	1417	0.81	0.51
	2	0.68	0.58–0.79	1422	0.81	0.46	0.65	0.54–0.76	1505	0.81	0.38
	1	0.67	0.57–0.78	1464	0.81	0.40	0.64	0.53–0.75	1516	0.83	0.33
Tanaka ^§^ 1	4	0.76	0.67–0.86	1413	0.83	0.66	0.75	0.65–0.85	1486	0.83	0.51
Second spot urine after waking	3	0.74	0.64–0.84	1451	0.81	0.58	0.72	0.61–0.82	1510	0.85	0.49
	2	0.75	0.65–0.85	1456	0.81	0.66	0.70	0.59–0.81	1540	0.81	0.49
	1	0.71	0.60–0.81	1695	0.81	0.40	0.72	0.62–0.82	1668	0.81	0.49

Abbreviations: AUC, area under the curve; CI, confidence interval; ROC, receiver operating characteristic; 24 h UC, 24-h urine collection; CO, cutoff value; Se, sensitivity; Spe, specificity. * Number of participants who deviate from the criterion based on a 12-day dietary survey using a mobile-based photographic dietary record system as the reference. ^†^ Cutoff values determined as thresholds maximizing specificity among those with a sensitivity >0.80. ^‡^ Urinary potassium excretion estimated using the Kawasaki equation. ^§^ Urinary potassium excretion estimated using the Tanaka equation.

## Data Availability

The datasets used in this study are available from the corresponding author upon reasonable request, as the informed consent obtained did not include provisions for data disclosure.

## Supplementary Materials

The following supporting information can be downloaded at: https://www.mdpi.com/article/10.3390/nu18050735/s1, Table S1. AUC (95% CI) of ROC curves for screening individuals with insufficient usual vegetable intake or total fruit and vegetable intake using urinary potassium excretion from 24-h UCs or spot urine samples across 4 days (additional intake criteria; *n* = 97).
