# Supplementary material for: Screening Performance for Insufficient Vegetable Intake in Young Adult Japanese Women: Spot Urinary Potassium Excretion as a Simple, Non-Invasive Marker
Source: Nutrients. 2026 Feb 25;18(5):735. doi: 10.3390/nu18050735 (PMC12986915; doi:10.3390/nu18050735)
Supplement: Supplementary file 1 [file nutrients-18-00735-s001.zip › nutrients-4135406-supplementary.pdf]

Table S1. AUC (95% CI) of ROC curves for screening individuals with insufficient usual vegetable intake or total fruit and vegetable intake using urinary potassium excretion from 24-h UCs or spot urine samples across 4 days (additional intake criteria; n = 97).

	Vegetables							Total fruit and vegetables						
	Criteria	n*	AUC	95%CI	CO†	Se	Spe	Criteria	n*	AUC	95%CI	CO†	Se	Spe
**24-h UC**	≥200g	82	0.66	0.50–0.81	1657	0.82	0.40	≥150g	23	**0.70**	**0.58**–0.81	1393	0.83	0.49
	≥250g	91	**0.70**	**0.50**–0.91	1783	0.89	0.50	≥250g	73	**0.73**	**0.61**–0.84	1521	0.81	0.50
	≥300g	96	0.48	0.38–0.58	2983	1.00	0.00	≥300g	87	**0.72**	**0.57**–0.87	1774	0.90	0.50
**Kawasaki‡**	≥200g	82	0.65	0.49–0.81	2094	0.84	0.33	≥150g	23	**0.78**	**0.69**–0.87	1681	0.83	0.64
Second spot urine after waking	≥250g	91	0.68	0.49–0.88	2180	0.88	0.33	≥250g	73	**0.76**	**0.64**–0.87	1905	0.81	0.58
	≥300g	96	0.59	0.49–0.69	2640	1.00	0.00	≥300g	87	**0.71**	**0.55**–0.86	2180	0.89	0.30
**Tanaka§ 3**	≥200g	82	0.63	0.48–0.79	1482	0.80	0.33	≥150g	23	**0.75**	**0.65**–0.85	1346	0.83	0.55
All 3 spot urine samples	≥250g	91	0.61	0.38–0.84	1520	0.86	0.17	≥250g	73	**0.73**	**0.61**–0.85	1474	0.85	0.50
	≥300g	96	**0.79**	**0.71**–0.87	1808	1.00	0.00	≥300g	87	0.67	0.51–0.84	1477	0.80	0.50
**Tanaka§ 2**	≥200g	82	0.58	0.41–0.74	1519	0.85	0.33	≥150g	23	**0.72**	**0.62**–0.83	1329	0.83	0.61
Randomly selected 2 of 3 samples	≥250g	91	0.54	0.29–0.78	1519	0.84	0.33	≥250g	73	0.67	0.54–0.80	1463	0.82	0.46
	≥300g	96	**0.86**	**0.80**–0.93	1536	0.86	1.00	≥300g	87	0.61	0.43–0.80	1519	0.84	0.30
**Tanaka§ 1**	≥200g	82	0.68	0.52–0.84	1518	0.84	0.40	≥150g	23	0.69	0.58–0.79	1344	0.83	0.54
Randomly selected 1 of 3 samples	≥250g	91	0.68	0.42–0.94	1518	0.82	0.50	≥250g	73	**0.73**	**0.60**–0.85	1441	0.81	0.46
	≥300g	96	**0.94**	**0.89**–0.99	1636	0.94	1.00	≥300g	87	**0.72**	**0.51**–0.92	1518	0.84	0.50
**Tanaka§ 1**	≥200g	82	0.63	0.49–0.78	1754	0.90	0.20	≥150g	23	**0.77**	**0.68**–0.87	1384	0.91	0.58
Second spot urine after waking	≥250g	91	0.64	0.45–0.84	1754	0.89	0.17	≥250g	73	**0.73**	**0.62**–0.85	1546	0.82	0.58
	≥300g	96	**0.73**	**0.64**–0.82	2028	1.00	0.00	≥300g	87	0.69	0.54–0.84	1790	0.91	0.20

Abbreviations: AUC, area under the curve; CI, confidence interval; ROC, receiver operating characteristic; 24-h UC, 24-h urine collection; CO, cutoff value; Se, sensitivity; Spe, specificity. * Number of participants who deviate from the criterion based on a 12-day dietary survey using a mobile-based photographic dietary record system as the reference. † Cutoff values determined as thresholds maximizing specificity among those with a sensitivity > 0.80. ‡ Urinary potassium excretion estimated using the Kawasaki equation. § Urinary potassium excretion estimated using the Tanaka equation.
